# Potential inappropriate medications and drug–drug interactions in adverse drug reactions in the elderly: a retrospective study in a pharmacovigilance database

**DOI:** 10.3389/fphar.2025.1546012

**Published:** 2025-04-08

**Authors:** Huaqiao Jiang, Yanhua Lin, Weifang Ren, Lina Lu, Xiaofang Tan, Xiaoqun Lv, Ning Zhang

**Affiliations:** ^1^ Department of Pharmacy, Jinshan Hospital, Fudan University, Shanghai, China; ^2^ Department of Nursing, Jinshan Hospital, Fudan University, Shanghai, China; ^3^ Department of Dermatology, Jinshan Hospital, Fudan University, Shanghai, China

**Keywords:** potentially inappropriate medicines, drug–drug interactions, elderly, adverse drug reactions, real-world

## Abstract

**Introduction:**

Potential inappropriate medications (PIMs) and potential drug–drug interactions (pDDIs) are important factors leading to adverse drug reactions (ADRs) in the elderly. This study aimed to evaluate the incidence and pattern of PIMs and pDDIs in the elderly based on a real-world pharmacovigilance database and identify the variables associated with them.

**Methods:**

This retrospective study evaluated PIMs and pDDIs by updated Beers criteria and Lexi-Interact online, respectively, using ADRs reported for those aged ≥65 years submitted between 2011 and 2023 from a real-world database of a tertiary care teaching hospital. Correlation factors were investigated by binary and multiple logistic regression analyses.

**Results:**

A total of 1,423 ADRs were included and involved 2,238 prescribed drugs; 54.11% of the total were men, and 23.47% were classified as serious. The most commonly implicated pharmacological group was antimicrobial agents. Aspirin and clopidogrel emerged as the drugs causing the majority of ADRs. PIMs were detected in 32.04% of all ADR reports. Aspirin and diclofenac were the most common active pharmaceutical ingredients involved, and gastrointestinal bleeding was the primary clinical manifestation of severe ADRs caused by PIMs or involved in PIM-related risk factors. Age, number of diagnosed diseases and prescribed drugs, ADR severity and preventability, hypertension, coronary heart disease, and arthritis were independent influencing factors of PIMs. Among 498 ADR reports with ≥2 prescribed drugs, 202 cases (14.20%) had pDDIs. Blood and hematopoietic organ and cardiovascular agents were the most commonly involved categories. The most frequent drug combinations in classes C, D, and X were aspirin–clopidogrel, aspirin–heparin, and potassium chloride–promethazine, respectively. The majority of pDDIs increased the risk of bleeding through pharmacodynamic mechanisms. The number of prescribed drugs and diagnosed diseases, ADR severity and preventability, stroke, diabetes, and coronary heart disease, along with PIM use, were independent predictors of pDDIs.

**Conclusion:**

The incidence of PIMs and pDDIs was found to be relatively high in the elderly, especially in the treatment of cardiovascular and cerebrovascular diseases and non-steroidal anti-inflammatory drugs (NSAIDs), and relevant factors have been identified. Healthcare institutions should reinforce the management of rational drug use in the elderly to mitigate the occurrence of PIMs and pDDIs, thereby enhancing medication safety.

## 1 Introduction

With the progress of society and the improvement of medical care, along with extended life expectancy, the aging process of the global population is accelerating. By 2050, the world's elderly population will rise from 9% in 2019 to 16%, while it will reach nearly 33% of the country's total population in China and enter a stage of severe aging that will persist for a long time (Dong and Zhang, 2017; Population, 2019). The elderly population represents a significant segment of healthcare consumers, and their medication usage patterns remain complex and challenging due to the presence of multiple chronic conditions. Consequently, the risk of potentially inappropriate medications (PIMs) and potential drug–drug interactions (pDDIs) increases, posing a significant threat to the health and well-being of the elderly and resulting in enormous economic impacts on the healthcare system (Pohl-Dernick et al., 2016).

The prevalence of PIMs and pDDIs in the elderly is well-documented. The global prevalence of potentially inappropriate prescribing is 13% to 35% (Chen et al., 2012), and the incidence of pDDIs in older adults ranges from 80.5% to 90.5% (Oliveira et al., 2021). These data are directly associated with significant morbidity, mortality, and healthcare resource consumption (Xing et al., 2019; Fralick et al., 2020). Numerous studies have been conducted in various healthcare settings, such as outpatient and emergency departments, internal medical wards, and community residences and nursing homes, to investigate these issues (Lao et al., 2013; Marinović et al., 2020; Roux et al., 2020; Anfinogenova et al., 2021; Kim et al., 2021; Očovská et al., 2023). However, a gap remains in our understanding of PIMs and pDDIs in the elderly based on adverse drug reaction (ADR) data. ADRs provide a unique opportunity to identify real-world patterns of medication use and their associated risks in this vulnerable population.

ADRs include unintended and undesired effects that occur when using a medication, and they offer a unique window into the safety and efficacy of drugs in actual use. They can range from mild and transient symptoms to severe and life-threatening conditions. In the elderly, ADRs are usually caused by PIMs and pDDIs due to the presence of multiple comorbidities, polypharmacy, and pharmacokinetic and pharmacodynamic processes that undergo significant alterations resulting from the physiological and cognitive changes associated with the aging process. It has been documented that 29.8% of all ADRs were considered caused by PIMs, and approximately 3% to 26% of hospital admission-related ADRs are due to pDDIs (Hedna et al., 2015; Aljadani and Aseeri, 2018). Therefore, analyzing pharmacovigilance data in the elderly population has the potential to provide valuable insights into PIMs and pDDIs, which can inform clinical decision-making and improve patient safety.

Currently, various clinical tools and algorithms have been developed to identify PIMs and pDDIs in the elderly, such as the best known tools: the American Geriatric Society (AGS) Beers Criteria (Panel, 2023), the START/STOPP criteria (O'Mahony et al., 2014), and the Priscus List (Holt et al., 2010). In this study, the Beers Criteria 2023, which is the most recently revised criteria for geriatric prescribing, was applied since it was the most used tool in the general population. Similarly, there are several databases for the identification of pDDIs, of which the Lexi-Interact software in the UpToDate online interaction program and Micromedex were most frequently used (Vitry, 2006; Bories et al., 2021). The pDDI tool can systematically analyze possible interactions between different drugs and provide an important basis for avoiding potential risks. Unfortunately, its simultaneous application in ADR data for the elderly is limited and rarely reported.

Therefore, the purpose of this study was to (i) determine the incidence and patterns of PIMs and pDDIs in the elderly based on real-world ADR data by employing the established Beers Criteria tools and Lexi-Interact software, and (ii) investigate the factors of PIMs and pDDIs in the elderly and their correlations.

## 2 Methods

### 2.1 Study design and data source

This study is a branch of our previous retrospective single-center observational study (Jiang et al., 2022). All ADR data collection, which included outpatient and hospitalization, was performed retrospectively in the National ADR surveillance system of Jinshan Hospital of Fudan University from 1 January 2011 to 31 December 2023. ADR reports were filled out in specific ADR report formats and submitted by healthcare professionals (such as doctors, pharmacists, and nurses) in paper or electronic form. The process for ADR review, evaluation, confirmation, and report submission was the same as in the past. Each report corresponds to one patient and can describe one or more ADRs. In this sub-study, elderly patients aged ≥65 exposed to at least one drugs suspected of causing ADRs were included; others were discarded.

Recorded in the ADR reports were demographic characteristics such as sex, age, diagnosis, prescribed drugs, drug combination, drug details, organ system involved in ADRs, ADR treatment and outcome, and type of reporter. According to the Anatomical Therapy Chemistry (ATC) classification of the World Health Organization (WHO), criminal drugs were classified pharmacologically. The systems organ classes (SOC) affected were coded based on the WHO adverse reaction terminology (WHO-ART), and the data were cross-checked by two clinically experienced investigators to ensure accuracy.

### 2.2 ADR report characterization

The definition of ADRs follows the WHO definition (Edwards and Aronson, 2000), and the assessment of causality is determined by the Naranjo algorithm (Naranjo et al., 1981), which classifies causality as doubtful (score ≤0), possible ADRs (score 1–4), probable ADRs (score 5–8), and definite ADRs (score ≥9). The severity level is based on the Hartwig scale, where a serious ADR is defined as any ADR that results in the need for intensive care, permanent harm to the patient, or patient death (Hartwig et al., 1992). The modified Schoumock and Thornton scales (GT and JP, 1992) were used to evaluate the preventability of ADRs. In this study, “definitely” and “probably” preventable are considered preventable.

### 2.3 PIM and pDDI identification

Based on the information provided in each ADR report, we applied the 2023 AGS-updated Beers criteria to the PIM classification of prescribed drugs in ADR reports whenever possible. It was not feasible to apply the START/STOP criteria in this study, as the required complete clinical information was not available in any of the reports. In each report, the dose and duration of treatment can be determined so that the PIM criteria relating to dose and duration of treatment can be utilized. When clinically relevant information is required, such as PIMs related to kidney function, these criteria cannot be employed unless they are documented in some reports. This standard applies to the information available in ADR reports; if the information is insufficient, a prescribed drugs cannot be identified as a PIM.

For ADRs caused by two or more prescribed drugs, pDDIs were identified by the software Lexi-Interact in the UpToDate online interaction program and were divided into five risk levels: X (avoid combination therapy), D (consider therapy modification), C (monitor therapy), B (no action needed), and A (no known interaction). The C, D, and X risk ratings always require user attention, so we uniformly considered them as serious pDDIs in this study. The probability, severity, preventability, and PIMs of ADRs were independently evaluated by two clinical pharmacists, and any disagreements were settled by discussion. Compound medications were calculated based on their main active ingredients.

### 2.4 Statistical analysis

Descriptive analysis was performed to describe the population and clinical characteristics of ADRs, PIMs, and pDDIs. Continuous data were described as medians with standard deviation (SD) or median with 25% to 75% interquartile range (IQR), while categorical data were described as proportions and frequencies. Fisher’s exact test and Pearson’s χ2 test were performed for categorical data, and the Mann–Whitney U test was performed for continuous data. Multivariate correlations between PIMs or pDDIs and some explanatory variables in ADRs were conducted by logistic regression models. Statistical analysis was performed using IBM SPSS Statistics version 25. A p-value <0.05 was considered statistically significant.

## 3 Results

### 3.1 ADR report characterization

**FIGURE 1:**
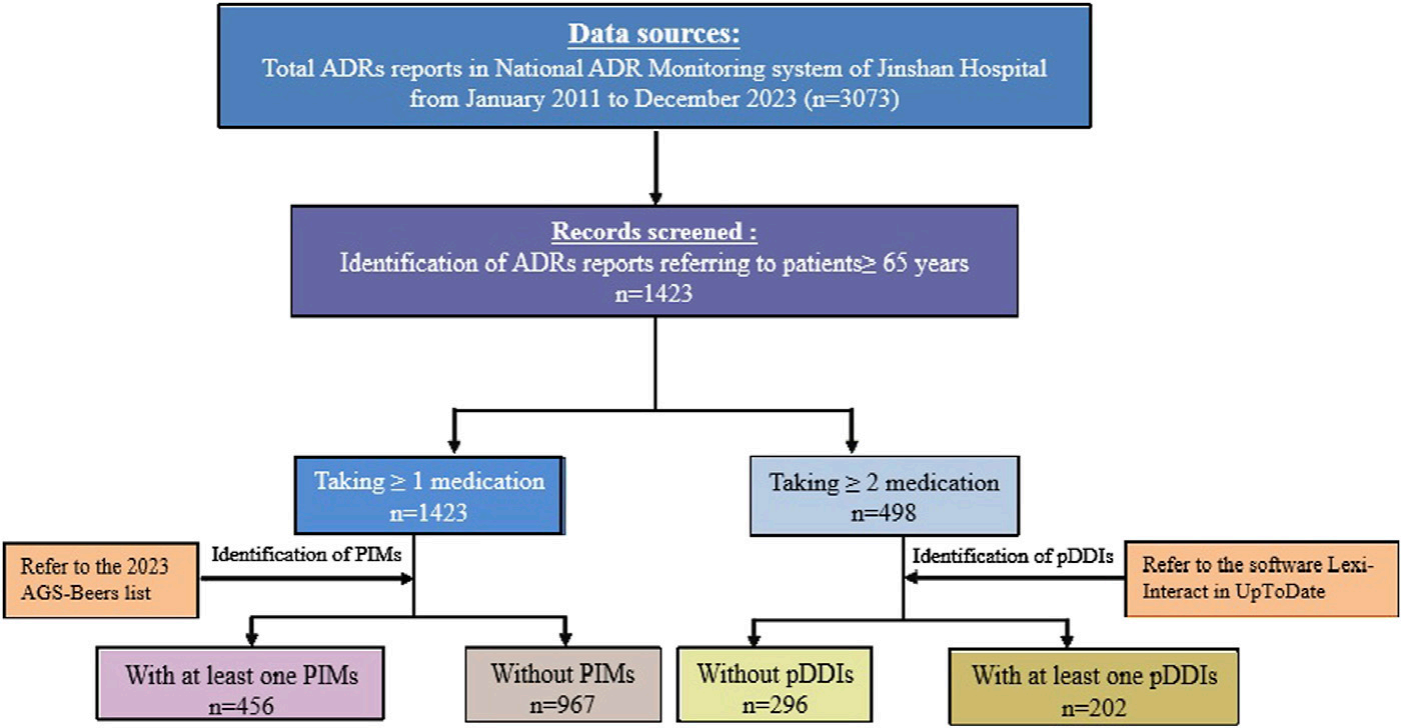
Flowchart depicting the study process.

A total of 3,073 ADRs were reported by healthcare professionals in our hospital over a period of 13 years. Following the exclusion of patients younger than 65 years, 1,423 (46.31%) ADR reports were eligible for the evaluation of PIMs use and/or pDDIs (Figure 1). The study population had a median age of 75 years (range 65–98; IQR 69-81), and 54.11% were men. Of the reports received, 23.47% were classified as serious, 78.15% were preventable, and 77.93% were reported by a pharmacist (Table 1).

**TABLE 1:** Characterization of elderly patients’ ADR reports.

Category	Number of ADRs (%)
Gender	
Male	770 (54.11)
Female	653 (45.89)
Age (years)	
65–74 years	693 (48.70)
75–84 years	541 (38.02)
≥85 years	189 (13.28)
Number of prescribed drugs	
1	925 (65.00)
2–3	417 (29.31)
≥4	81 (5.69)
Number of diagnosed diseases	
1	640 (44.97)
2–4	580 (40.76)
≥5	203 (14.27)
Severity assessment	
Mild	349 (24.53)
Moderate	740 (52.00)
Severe	334 (23.47)
Causality assessment	
Definite	4 (0.28)
Probable	682 (47.93)
Possible	737 (51.79)
Preventability	
Definitely/probably preventable	1112 78.15)
Unpreventable	311 (21.85)
Reporter occupation	
Physician	314 (22.07)
Pharmacist	1109 (77.93)

Within the total number of reports, 2,238 prescribed drugs (min. 1; max. 10) containing 468 different active substances were implicated, with an average of 1.57 ± 1.01 drugs implicated per report. As shown in Table 2, the major implicated pharmacological groups in the reports were systemic antimicrobial agents (n = 432/19.30%), cardiovascular agents (n = 410/18.33%), blood and blood forming organ agents (n = 384/17.17%), and alimentary tract and metabolism agents (n = 255/11.39%). However, the two drug categories that most commonly resulted in serious ADRs were blood/blood-forming organ agents and cardiovascular agents. Aspirin and clopidogrel were the top two prescription drugs for both total and severe ADRs.

**TABLE 2:** Pharmacological groups according to the WHO-ATC code and their pattern in ADRs.

Pharmacological groups	Number of ADRs	ADR frequency (%)	Number of severe ADRs (%)	Number of PIMs (%)	Number of severe pDDIs (%)
Cardiovascular system	454	410 (18.33)	126 (30.73)	126 (30.73)	181 (44.15)
Blood and blood-forming organs	203	384 (17.17)	140 (36.46)	161 (41.93)	229 (59.64)
Musculoskeletal system	168	180 (8.04)	87 (48.33)	137 (76.11)	79 (43.89)
Alimentary tract and metabolism	205	255 (11.39)	55 (21.57)	48 (18.82)	42 (16.47)
Traditional Chinese medicine	105	112 (5.0)	43 (38.39)	6 (5.36)	41 (36.61)
Anti-infective agents for systemic use	330	432 (19.3)	76 (17.59)	2 (0.46)	32 (7.41)
Respiratory system	60	72 (3.22)	9 (12.50)	10 (13.89)	21 (29.17)
Nervous system	122	135 (6.03)	30 (22.22)	46 (34.07)	20 (14.81)
Systemic hormonal preparations excluding sex hormones and insulin	74	87 (3.89)	17 (19.54)	4 (4.60)	19 (21.84)
Antineoplastic and immunomodulating	95	129 (5.76)	15 (11.63)	0	13 (10.08)
Dermatologicals	1	2 (0.09)	0	0	1 (50)
Various	29	31 (1.39)	5 (16.13)	0	0
Genito-urinary system and sex hormones	7	7 (0.31)	1 (14.29)	3 (42.86)	0
Antiparasitic products, insecticides, and repellents	1	1 (0.04)	0	0	0
Sensory organs	1	1 (0.04)	0	0	0
Total^※^		2,238 (100%)			

^※^ Each ADR may have multiple prescribed drugs, so the total number of implicated drugs exceeds the ADRs. WHO-ATC, WHO, anatomical therapy chemistry classification; ADR, adverse drug reaction.

In total, 1,611 ADRs were identified (each report could contain more than one ADR from the same SOC). Table 3 displays the most commonly reported SOC, with “gastro-intestinal system disorders” being the most frequent, identified in 29.11% of the reports, followed by “skin and subcutaneous tissue disorders,” “liver and biliary system disorders,” and “platelet, bleeding and clotting disorders.” Gastrointestinal bleeding was the most common clinical manifestation.

**TABLE 3:** Organs/systems involved in ADRs according to WHO-ART classification during 2011–2023.

Organs/systems	Clinical manifestations or symptoms	Frequency (%)
Gastro-intestinal system disorders	Nausea, vomiting, abdominal pain, diarrhea, flatulence, melena, gastrointestinal bleeding, hematemesis, etc.	469 (29.11)
Skin and appendages disorders	Itching, urticaria, rash, maculopapular rash, erythema, etc.	221 (13.72)
Liver and biliary system disorders	Abnormal liver function, jaundice, elevated liver enzymes, cholestatic hepatitis, biliary cirrhosis	221 (13.72)
Platelet, bleeding, and clotting disorders	Bone marrow suppression, thrombocytopenia, coagulopathy, hematemesis, etc.	180 (11.17)
Body-as-a-whole general disorders	Fatigue, allergic reactions, chills	122 (7.57)
Central and peripheral nervous system disorders	Dizziness, headache, coma, grand mal seizure, etc.	97 (6.02)
Metabolic and nutritional disorders	Electrolyte abnormalities, hyperuricemia, increased blood lactic acid, hypokalemia; hyponatremia, hyperkalemia, hypoglycemia, hyperglycemia, etc.	67 (4.16)
Urinary system disorders	Hematuria, abnormal renal function, urinary retention	46 (2.86)
Respiratory system disorders	Dyspnea, asthma, cough	36 (2.23)
Cardiovascular disorders, general	Hypotension, hypertension	35 (2.17)
Application site disorders	Phlebitis, skin necrosis	27 (1.68)
Psychiatric disorders	Circulatory psychotic reactions, insomnia, manic reactions, sleep disorders	23 (1.43)
Heart rate and rhythm disorders	Palpitations, tachycardia, bradycardia, cardiac arrest, arrhythmias, atrioventricular block	17 (1.06)
Musculoskeletal system disorders	Myasthenia, myalgia, muscle bleeding, arthralgia, lower limb spasm	14 (0.87)
White cell and res disorders	Leukopenia, leukopenia, granulocytopenia and granulocytopenia	13 (0.81)
Endocrine disorders	Male breast pain, non-specific endocrine disease, thyroiditis, hyperparathyroidism	9 (0.56)
Vision disorders	Conjunctival hemorrhage	6 (0.37)
Red blood cell disorders	Anemia	5 (0.31)
Resistance mechanism disorders	Decreased IgG4, systemic lupus erythematosus syndrome, fungal infection	3 (0.19)
Sum^※^		1611 (100%)

^※^ Some ADRs involve multiple organs or systems from the same SOC. WHO-ART, WHO adverse reaction terminology; ADR, adverse drug reaction.

### 3.2 Characteristics of PIMs identified in ADR reports

Of the 456 ADR reports (32.04% of total ADR reports) that involved 543 medicines, a total of 692 PIMs were detected. Of these, 63.38% of the ADR reports were taking one PIM, and 0.22% were taking nine PIMs. The categories of PIMs were based on the anatomical group (Table 2), and it was observed that of the 180 implicated medicines belonging to the musculoskeletal system, 76.11% were considered PIMs. In contrast, the cardiovascular agents group with the highest number of drugs involved contained 410 implicated medicines, of which 30.73% were PIMs. Moreover, the analysis of the 255 medicines belonging to the alimentary tract and metabolism and the 384 medicines belonging to the blood and blood-forming organs revealed that 18.82% and 41.93%, respectively, were classified as PIMs.

**FIGURE 2:**
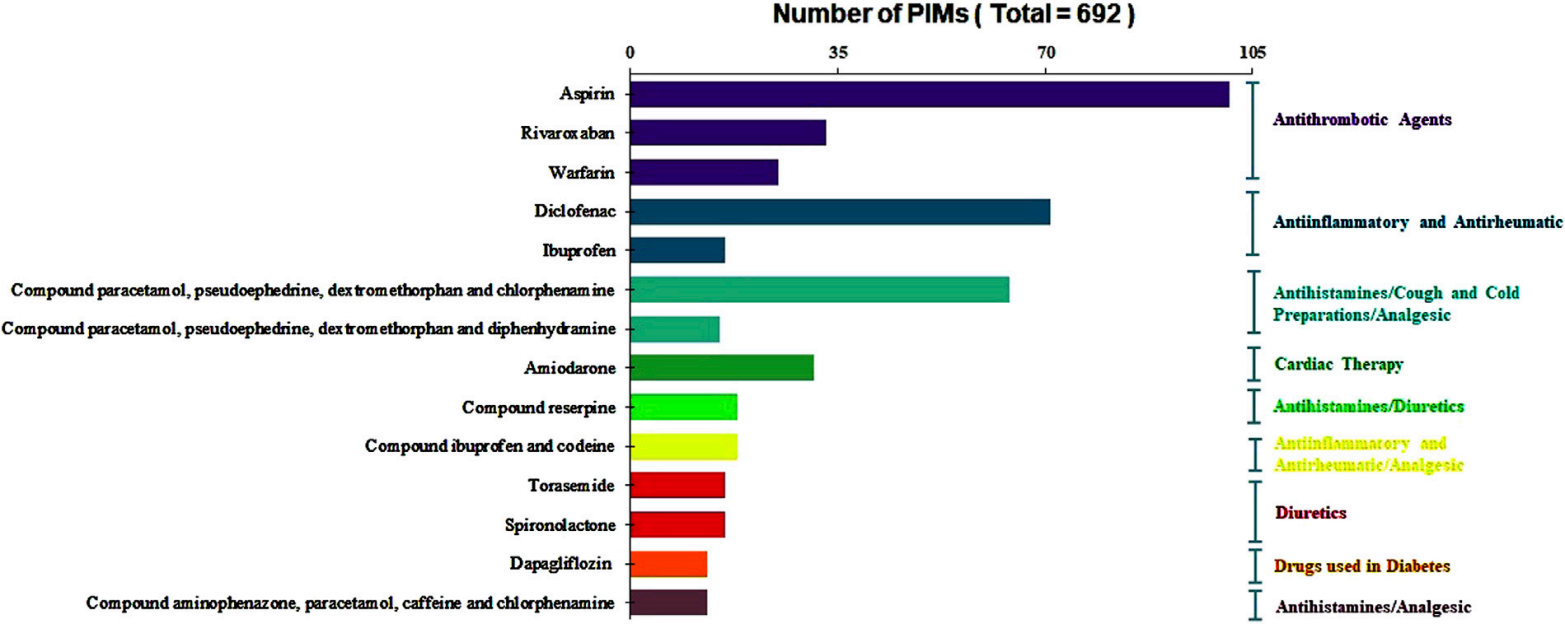
Distribution of the ten most commonly prescribed of PIMs and their corresponding ATC subgroup classifications.

Of the individual drugs, aspirin, diclofenac, compound paracetamol, pseudoephedrine hydrochloride, dextromethorphan and chlorphenamine maleate, and rivaroxaban were the top four PIMs, accounting for 14.60%, 10.26%, 9.25%, and 4.77% of the total number of PIMs, respectively. The ten most commonly prescribed PIMs and their corresponding ATC subgroup classifications are illustrated in Figure 2. It is noteworthy that several compound preparations contain chlorpheniramine maleate and paracetamol, such as compound paracetamol, pseudoephedrine hydrochloride, dextromethorphan and chlorphenamine maleate, compound pseudoephedrine hydrochloride, compound paracetamol, aminophenazone, caffeine, and chlorphenamine maleate. Additionally, compound pseudoephedrine hydrochloride also contains the active ingredient of dextromethorphan.

Of the 456 ADRs containing PIMs, 285 cases (62.50%) involving drug-related PIMs, 168 (36.84%) related to PIMs in older adults due to drug–disease or drug–syndrome interactions that may exacerbate the disease or syndrome, and 127 cases (27.85%) should be used with caution. There was one case (0.02%) of potentially clinically important drug–drug interactions that should be avoided and 37 PIM warning cases (8.11%) due to renal insufficiency. The most frequent active drug ingredient with potential drug-related inappropriateness was chlorphenamine maleate, and the highest frequency of potential disease-related inappropriateness was in patients with a history of gastric or duodenal ulcer disease using aspirin, a cardio-cerebrovascular therapy with a similar mechanism to non-COX-2-selective nonsteroidal anti-inflammatory drugs (NSAIDs) (Supplementary Table S1).

We further investigated whether the reported ADRs were caused by PIMs or involved PIM-related risk factors. The results indicated that among 456 patients, 242 cases were attributed to PIMs or had PIM-related risk factors, and 106 were severe ADRs, accounting for 23.25%. Aspirin and diclofenac were the most commonly active pharmaceutical ingredients involved, gastrointestinal bleeding was the primary clinical manifestation of severe ADRs caused by PIMs or involving PIM-related risk factors (Supplementary Table S2).

Binary logistic regression was performed to assess the predictors of PIMs in geriatric patients; the results are summarized in Table 4. Age, number of sprescribed drugs, severity of ADRs, preventability of ADRs, and certain chronic diseases such as hypertension, arthritis (all p < 0.001), and coronary heart disease (p = 0.003) were significantly associated with PIMs. No association was found between PIMs and gender, the number of diagnosed diseases, diabetes, and history of stroke (all p > 0.05). Multivariate logistic regression analysis indicated that age (OR 1.03, 95% CI 1.01–1.05, p = 0.003), number of diagnosed diseases (OR 0.78, 95% CI 0.72–0.85, p < 0.001), number of prescribed drugs (OR 1.42, 95% CI 1.25–1.62, p < 0.001), severity of ADRs (OR 2.42, 95% CI 2.0–2.93, p < 0.001), preventability of ADRs (OR 1.72, 95% CI 1.22–2.44, p = 0.002), hypertension (OR 2.32, 95% CI 1.69–3.17, p < 0.001), coronary heart disease (OR 1.72, 95% CI 1.21–2.45, p = 0.003), and arthritis (OR 4.29, 95% CI 2.58–7.11, p < 0.001) were independent factors for PIMs in older adults.

**TABLE 4:** Variable analysis to identify independent factors associated with PIMs and pDDIs through elderly patients’ ADR reports.

Characteristics	Bivariate analysis		Multivariate analysis			Bivariate analysis			Multivariate analysis	
	Patients with PIMs (N = 456)	Patients without PIMs (N = 967)	p value	Or (95% CI)	p value	Patients without pDDIs (N = 296)	Patients with pDDIs (N = 202)	p value	Or (95% CI)	p value
Age, years, median(IQR) ^a^	76 (71–81)	74 (69–80)	<0.001^b^	1.03 (1.01–1.05)	0.003	75 (69–81)	76 (70–82)	0.116	1.01 (0.98–1.04)	0.616
Gender										
Male	255	515	0.347	0.96 (0.75–1.24)	0.772	188	123	0.553	1.07 (0.68–1.68)	0.766
Female	201	452				108	79			
Number of prescribed drugs (median(IQR)) ^a^	1 (1–2)	1 (1–2)	<0.001	1.42 (1.25–1.62)	<0.001	2 (2–2)	3 (2–4)	<0.001	2.11 (1.64–2.71)	<0.001
Number of diagnosed diseases (median(IQR)) ^a^	2 (1–3)	2 (1–3)	0.942	0.78 (0.72–0.85)	<0.001	2 (1–4)	2 (1–4)	0.217	0.79 (0.70–0.90)	<0.001
Severity assessment			<0.001	2.42 (2.0–2.93)	<0.001			<0.001	1.48 (1.04–2.13)	0.032
Mild	50	299				49	15			
Moderate	223	517				177	99			
Severe	183	151				70	88			
Preventability			<0.001	1.73 (1.22–2.44)	0.002			0.001	2.02 (0.94–4.35)	0.071
Preventable	402	710				248	190			
Unpreventable	54	257				48	12			
History of stroke	91	197	0.855	1.04 (0.75–1.42)	0.83	60	57	0.04	2.51 (1.49–4.21)	0.001
Hypertension	188	295	<0.001	2.32 (1.69–3.17)	<0.001	112	90	0.134	0.98 (0.59–1.64)	0.946
Diabetes	74	158	0.958	1.30 (0.91–1.84)	0.151	36	39	0.029	2.18 (1.81–4.01)	0.013
Coronary heart disease	91	133	0.003	1.72 (1.21–2.45)	0.003	38	68	<0.001	4.95 (2.70–9.07)	<0.001
Arthritis	52	31	<0.001	4.29 (2.58–7.11)	<0.001	11	18	0.015	2.48 (1.01–6.09)	0.048
PIM use (No. (%))	NA	NA				88	137	<0.001	2.12 (1.05–4.30)	0.037

PIMs, potentially inappropriate medications; pDDIs, potential drug–drug interactions; IQR, interquartile range; CI, confidence interval; OR, odds ratio.

^a^ Mann–Whitney U test. All others were performed using Chi chi-square test.

### 3.3 Characteristics of pDDIs identified in ADR reports

Among the 1,423 ADR reports, 498 (35.0%) involved more than two drugs suspected of causing ADRs. pDDIs were observed in 202 ADR reports (14.20% of total ADR reports), and 186 ADRs involved severe pDDIs, of which 127 had one serious pDDI and 59 contained two or more serious pDDIs. There were 339 serious pDDI drug combinations: 236 were level C in 141 ADRs, 89 were level D in 64 ADRs, and 14 were level X in 11 ADRs. The maximum number of severe pDDIs was 15, which were detected in one patient. Overall, drugs from the blood and blood-forming organs were implicated in 33.78% of serious pDDIs. Cardiovascular agents were the second most represented therapeutic/pharmacological group (26.70% of serious pDDIs). The detailed pharmacoanatomical groups involved in severe pDDIs are noted in Table 2.

**FIGURE 3:**
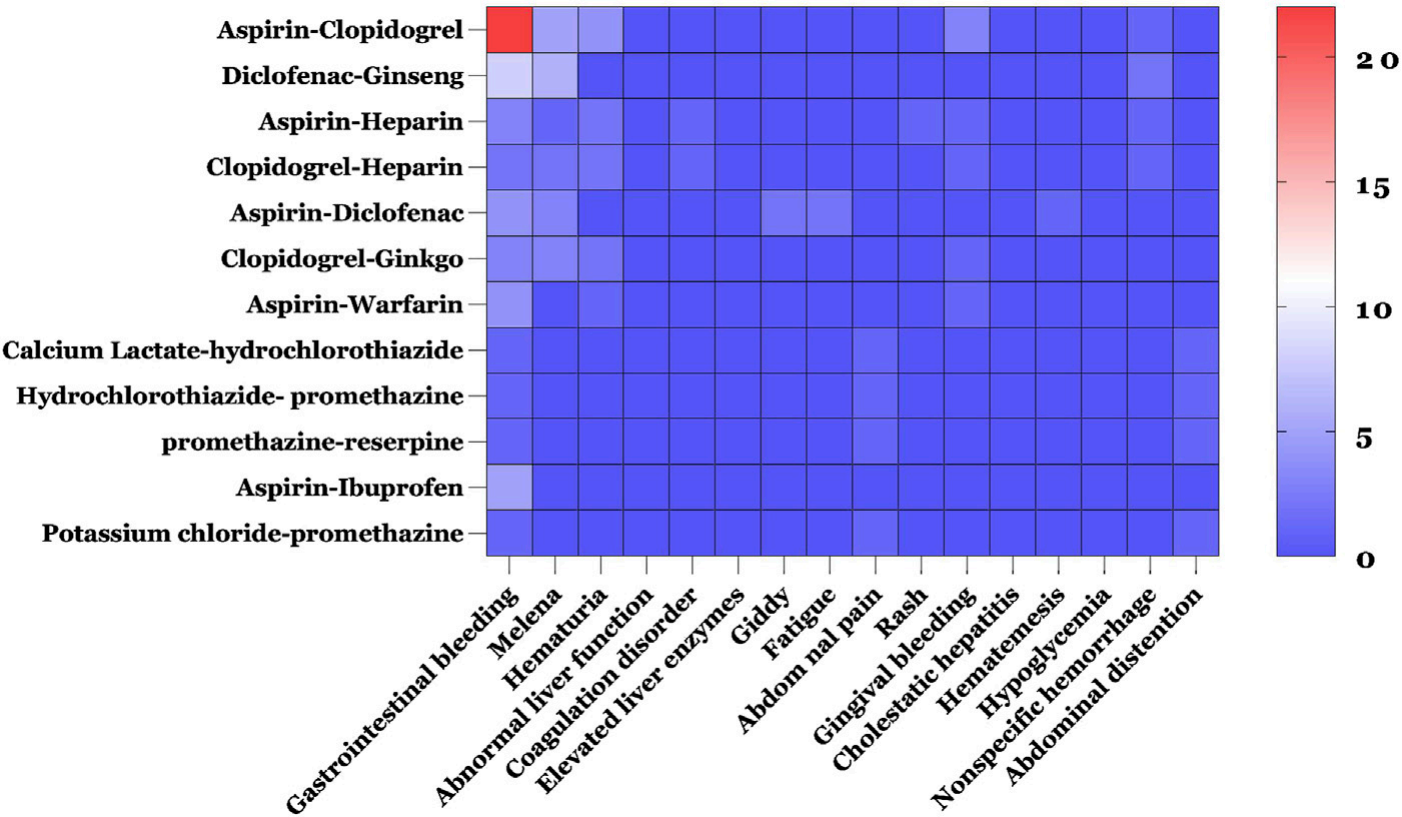
Heat map of the relationship between the top ten adverse reactions and pDDI frequency ≥5. Blue and red indicate the lowest and highest frequencies of different ADRs caused by pDDIs.

Table 5 shows the drug combinations with frequency ≥4. The most common drug combinations of pDDIs corresponding to levels C, D, and X were aspirin–clopidogrel, aspirin–heparin, and potassium chloride–promethazine, which accounted for 9.44%, 2.95%, and 1.47% of all severe pDDIs, respectively. Of the 186 ADR reported, a large proportion (175/186) of patients experienced drug interactions through the pharmacodynamic (PD) action addition mechanism, with the most frequent potential clinical outcome being increased risk of bleeding. To investigate the relationship between potential DDIs and ADRs, the ten most adverse reactions and pDDI frequencies ≥5 were analyzed. The results revealed that among the top ten ADRs, bleeding-related reactions were the most prevalent, with gastrointestinal bleeding ranking first, followed closely by melena. The potential DDI that caused the most gastrointestinal bleeding was aspirin–clopidogrel, followed by diclofenac–ginseng (Figure 3).

**TABLE 5:** Drug combinations with pDDIs ≥4.

Risk rating	Drug pairs	Mechanism of interaction	Potential clinical consequences	Evidence	Severity	Frequency (%, total = 339)
C	Aspirin–clopidogrel	PD	Increased effect of antiplatelet	Fair	Moderate	32 (9.44%)
C	Diclofenac–ginseng	PD	Increased risk of bleeding	Fair	Moderate	12 (3.54%)
D	Aspirin–heparin	PD	Increased risk of bleeding	Good	Moderate	10 (2.95%)
D	Clopidogrel–heparin	PD	Increased effect of anticoagulant	Good	Moderate	10 (2.95%)
D	Aspirin–diclofenac	PK/PD	Increased risk of bleeding	Good	Moderate	7 (2.06%)
D	Aspirin–warfarin	PD	Increased effect of anticoagulant	Excellent	Major	6 (1.77%)
C	Clopidogrel–ginkgo	PD	Increased risk of bleeding	Fair	Moderate	6 (1.77%)
D	Aspirin–ibuprofen	PK/PD	Increased risk of bleeding	Good	Moderate	5 (1.47%)
X	Potassium chloride–promethazine	PD	Increased effect of ulcerogenic	Fair	Moderate	5 (1.47%)
C	Hydrochlorothiazide– promethazine	PK	Increased diuretics serum concentration	Fair	Moderate	5 (1.47%)
C	Hydrochlorothiazide–calcium lactate	PK	Increased calcium salt serum concentration	Good	Moderate	5 (1.47%)
C	Promethazine–reserpine	PD	Increased adverse/toxic effect of CNS depressants	Good	Moderate	5 (1.47%)
D	Aspirin–ginkgo	PD	Increased effect of anticoagulant	Fair	Major	4 (1.18%)
D	Aspirin–ticagrelor	PD	Increased effect of antiplatelet	Fair	Major	4 (1.18%)
D	Aspirin–rivaroxaban	PD	Increased risk of bleeding	Fair	Major	4 (1.18%)
C	Aspirin–ginseng	PD	Increased risk of bleeding	Fair	Moderate	4 (1.18%)
D	Rivaroxaban-–clopidogrel	PD	Increased risk of bleeding	Fair	Major	4 (1.18%)

PD, pharmacodynamics; PK, pharmacokinetics; CNS, central nervous system.

We also conducted a check on the consistency between the reported ADRs and the potential clinical consequences of pDDIs. This showed that 135 reported ADRs were consistent with the potential clinical consequences of serious pDDIs, accounting for 27.11% of the ADR reports with more than one prescribed drugs, and 91 were bleeding-related ADRs. Of these, 62 reports were serious ADRs. The most frequent drugs involved in serious ADRs caused by DDIs were aspirin (n = 53) and clopidogrel (n = 30), and the great majority of serious ADRs caused by DDIs were associated with gastrointestinal bleeding. Similarly, the most common drug combinations of serious ADRs caused by DDIs corresponding to levels C, D, and X were aspirin–clopidogrel, aspirin–heparin, and potassium chloride–promethazine (Supplementary Table S3).

In the univariable analysis of pDDIs, the number of prescribed drugs (p < 0.001), severity of ADRs (p < 0.001), preventability of ADRs (p = 0.001), stroke history (p = 0.04), diabetes (p = 0.029), coronary heart disease (p < 0.001), arthritis (p = 0.015), and the PIMs used (p < 0.001) were significantly associated with pDDIs. In the multivariate analysis, variables other than age, sex, hypertension, and preventability of ADRs remained significant (Table 4).

## 4 Discussion

This study revealed that over 46% of patients who experience ADRs were elderly, highlighting the high susceptibility of older adults to ADRs owing to polypharmacy, age-related physiological changes, and comorbidities (Davies and O'Mahony, 2015; Zazzara et al., 2021). In terms of the incidence of ADRs, there may be significant differences between male and female patients due to factors such as body mass index, fat composition, hormonal effects, drug sensitivity, or genetic differences in enzyme levels. However, the incidence of ADRs was slightly higher in male than in female patients, consistent with previous reports (Dubrall et al., 2020; Magro et al., 2021).

In this study, 23.47% of ADRs were classified as severe. Severe ADRs, as a major concern for public health, were a contributing factor to unplanned hospitalizations, increased morbidity, mortality, and healthcare costs (Schurig et al., 2018; Monteiro et al., 2020). The finding that 78.15% of ADRs were preventable was much higher than the results of studies conducted in Jordan and Canada, which showed that 44.7% and 64.1% of ADRs were preventable, respectively (Al Damen and Basheti, 2019; Woo et al., 2020). The predictable and preventable rates of ADRs were reported in the literature as 74.3%, 90.2%, and 8.6% to 62.8%, respectively (Lisha et al., 2017). The high percentage of preventable ADRs indicates that there is significant room for improvement in medication safety. Preventable ADRs often result from inadequate monitoring, communication breakdowns, inappropriate prescribing, and reassessment after medication changes (Al Damen and Basheti, 2019; Woo et al., 2020). The involvement of pharmacists in reporting ADRs (77.93% of reports) is particularly noteworthy, which underscores their importance in medication safety and can provide valuable insights into ADRs. Pharmacists are uniquely positioned to identify and mitigate ADRs through their knowledge of pharmacology, drug interactions, and patient medication history, being involved in the assessment of a wider range of ADRs, more complex reactions, or being more thorough in information gathering and evaluation (Anne et al., 2012; Aung et al., 2018; SL et al., 2020). Therefore, strategies such as improved communication and collaboration among healthcare professionals, standardized prescribing practices, incorporating comprehensive medication reviews and timely interventions by pharmacists, and utilizing clinical decision support systems could help reduce preventable ADRs.

The most common pharmacological group was systemic antimicrobial drugs, accounting for 19.30% of the total ADRs in our study; this is inconsistent with previous findings showing that cardiovascular drugs are among the most popularly prescribed medications in older adults and were associated with a high risk of ADRs (Sikdar et al., 2012; Doherty et al., 2023). Compared to the antihypertensive drugs commonly used in the cardiovascular system in these studies, aspirin and clopidogrel—typically prescribed for secondary prevention of cardiovascular and cerebrovascular diseases related to blood and blood-forming organs—emerged as the two prescription drugs most associated with ADRs, both severe and total, in our study. Won et al. (2022) showed that the most frequent ADR clinical manifestations among the elderly were skin lesions due to age-related pharmacokinetic and pharmacodynamic changes, skin changes, gastrointestinal problems, abnormal hematologic findings, and dizziness—in descending order of frequency. However, in contrast, our study manifests that the system most frequently affected by ADRs was the gastrointestinal system, accounting for 29.11%, and the most common clinical manifestation was gastrointestinal bleeding, likely related to the antiplatelet effects of these drugs and the predominant oral formulation. Oral drugs need to be absorbed through the digestive tract to enter the body, affecting the gastrointestinal system. A gastrointestinal reaction is obvious and easy to observe. Additionally, the extensive use of NSAIDs or medications containing these ingredients increases the risk of bleeding and liver injury, resulting in the frequent occurrence of related clinical symptoms. Thus, careful monitoring and dose adjustment in older patients receiving antiplatelet therapy are necessary.

In our research, approximately 32.04% of patients were prescribed PIMs, similar to a study in a Chinese elderly population (32.16%) (Li et al., 2021). A study of PIMs in the elderly through the French pharmacovigilance database showed that the prevalence of PIMs was 7.3%, much lower than our results (Montastruc et al., 2014). However, it was 46.5% and 94% in nursing homes and internal medical wards, respectively, which far exceeded ours (Lao et al., 2013; Perpétuo et al., 2021). This variation in PIM incidence between studies may be due to employing a different setting, study design, or evaluation tools. The high prevalence of PIMs is concerning, as it is associated with increased morbidity, mortality, and healthcare costs in older adults. A systematic review showed that the prevalence of PIMs in older outpatients ranged from 1.3% to 95.2%, and the most common PIMs were benzodiazepines (Tian et al., 2023). Nevertheless, PIMs in the musculoskeletal system accounted for the highest proportion of the corresponding pharmacological group in this research, and NSAID drugs dominated, suggesting that NSAIDs, which are commonly prescribed for musculoskeletal conditions, are often inappropriately used in older adults as a consequence of the variety of diseases in the elderly and the higher prevalence of chronic non-communicable diseases such as pain. Furthermore, overlapping use and abuse of similar antipyretic and analgesic drugs were also prevalent. The inappropriate use of NSAIDs in this population can lead to gastrointestinal bleeding, renal impairment, and cardiovascular events, necessitating special attention. As a single drug, aspirin was the most frequently prescribed medication for PIMs and PIM-induced ADRs or those involving PIM-related risk factors, which is consistent with Chinthalapudi et al. (2022).

The identification of PIMs also revealed potential drug-related and disease-related inappropriateness. For example, chlorphenamine maleate, a common antihistamine ingredient frequently associated with potential drug-related inappropriateness, may be linked to the widespread use of compounded formulations containing the same drug ingredient in the elderly in China. Meanwhile, aspirin use in patients with a history of gastric or duodenal ulcer disease represented a high risk of disease-related inappropriate medication. This may perhaps be one of the reasons for the frequent occurrence of bleeding and the most serious adverse reactions involving blood and blood-forming organ drugs in our study. Thus, it underscores the importance of considering both drug–drug and drug–disease interactions when prescribing medications to older adults.

Logistic regression analyses identified several independent factors associated with PIMs in older adults. Age and number of drugs are independent factors influencing PIMs, which have been confirmed in other studies (Alhawassi et al., 2019; Li et al., 2021; Tian et al., 2022). The presence of certain chronic conditions such as hypertension, arthritis, and coronary heart disease in older patients predicted the increased chance of PIMs. Multiple studies have demonstrated a significant association between PIMs and certain chronic conditions such as cardiovascular disease, diabetes, osteoporosis, and the increased number of chronic diseases (TJ et al., 2013; Alhawassi et al., 2019). There were differences observed in the p-value of the number of diagnosed diseases in univariate and multivariate regression. This could be affected by other factors, such as age and the number of prescribed drugs, but further research is needed to confirm this. We also found a significant association between ADR severity, ADR preventability, and PIMs, which may be an interesting finding. These findings also help us understand the factors associated with PIMs and highlight the need for comprehensive drug reviews, especially in patients with multiple comorbidities.

The detection of pDDIs in 14.20% of the total patients is another concern. Drug interactions can result in decreased drug efficacy, increased toxicity, and ADRs (Bettonte et al., 2022). The prevalence of DDIs is variable. Studies conducted in hospitalized patients in different clinical settings have shown it to range from 8.34% to 100%, while it varies from 80.5% to 90.5% in studies conducted in geriatric wards (LM et al., 2021). The reasons for the differences in DDI prevalence were related to the research objects included in each study, the year and time of the research, the sample size, and methodological differences, especially the methods and/or software applied to identify DDIs.

The finding that blood and blood-forming organs were most frequently implicated in serious pDDIs aligns with their extensive use in older adults. Aspirin and clopidogrel were commonly employed drugs for the secondary prevention of cardiovascular and cerebrovascular disease treatment. Their combined application for this purpose was also recommended by clinical guidelines and had reached expert consensus. However, they can produce pharmacodynamic interactions that increase the risk of bleeding, which was also the primary clinical outcome observed in this study. In addition, the most common drug combinations associated with severe pDDIs or serious ADRs caused by DDIs involved antiplatelet and anticoagulant agents such as aspirin–clopidogrel and aspirin–heparin, with bleeding being the primary clinical manifestation. The literature indicates that the most common medications causing DDIs are aspirin and clopidogrel, with gastric bleeding being the most frequent clinical manifestation. When a fatal ADR occurred, it was usually caused by bleeding, with the most common cause being the combination of antithrombotic/anticoagulant drugs and NSAID drugs (Létinier et al., 2021; Magro et al., 2021; Mar et al., 2022). Therefore, careful monitoring and dose adjustment were necessary when combining blood and blood-forming organ drugs. Similarly, the second-most representative medicine in severe pDDIs was cardiovascular drugs, suggesting that the combination of these drugs also needs close attention.

An association between pDDIs and various independent variables, such as the quantity of prescribed drugs, the severity of ADRs, the preventability of ADRs, and chronic diseases, was found. Unlike other studies, we also found that PIMs were significantly associated with pDDIs and were an independent influencing factor (Lao et al., 2013), further underscoring the complexity of medication management in older adults and emphasizing the necessity for comprehensive medication reviews and utilizing clinical decision support systems to identify and mitigate pDDIs.

In summary, to address the clinical implications of PIMs and DDIs, particularly concerning the increased risk of bleeding with the combined use of aspirin and clopidogrel in older adults, several strategies can be implemented to better avoid or prevent these risks. First, healthcare professionals should prioritize comprehensive medication reviews, especially for patients on multiple medications, to identify PIMs and DDIs. Utilizing clinical decision support systems can aid in this process by flagging potential drug interactions and inappropriate medications for older adults. For the specific combination of aspirin and clopidogrel, careful monitoring of bleeding risk is essential, and it should include regular assessment of platelet function, coagulation parameters, and close monitoring for signs/symptoms of bleeding. Dose adjustments or alternative therapies should be considered in patients with a history of gastrointestinal bleeding, ulcers, and renal impairment.

Several controllable factors stand out from the risk factor analysis that can be used for clinical prevention and evaluation of PIMs and DDIs. Age, the number of prescribed drugs, and the presence of certain chronic conditions were independent influencing factors associated with PIMs and DDIs. Therefore, targeting these factors through tailored medication management plans, dose adjustments based on renal function, and avoiding unnecessary polypharmacy can help mitigate risks.

Additionally, improving communication and collaboration among healthcare professionals is crucial. Ensuring that all members of the healthcare team are aware of a patient’s medication regimen, potential interactions, and risk factors can help prevent errors and adverse events.

Finally, patient education and involvement in their medication management should not be overlooked. Providing patients and caregivers with information about their medications, potential side effects, and the importance of reporting any symptoms can empower them to take an active role in their healthcare and help identify potential issues early.

While this study provides valuable insights into the patterns and characteristics of ADRs, PIMs, and pDDIs in older adults, it has several limitations that should be acknowledged. First, the data were collected retrospectively and relied on voluntary reporting by healthcare professionals, which may be subject to reporting bias. Second, the study focused only on ADRs reported in our hospital, limiting the generalizability of our findings to other settings. Future research could explore the implementation of interventions, such as the assessment of medication reviews, clinical decision support systems, and alternative non-pharmacological therapies, to mitigate ADRs, PIMs, and pDDIs in the elderly population. Third, the mutual influence of different factors leads to the difference in p-values in univariate and multivariate regression analyses, which needs further attention in future studies. In addition, studies comparing ADR rates across different healthcare settings could provide valuable insights into the generalizability of our findings.

## 5 Conclusion

In conclusion, this study provides a comprehensive and in-depth analysis of ADRs, PIMs, and pDDIs in elderly patients in our hospital. The high proportion of preventable ADRs, PIMs, and pDDIs highlights the need for improved medication management in this vulnerable population. Strategies such as regular medication reviews, the application of clinical decision support systems, and the promotion of alternative non-pharmacological therapies could help minimize ADRs and improve medication safety in older adults. Further research is needed to evaluate the effectiveness of these interventions and to identify additional strategies to improve medication safety in geriatric patients.

## Data Availability

The raw data supporting the conclusions of this article will be made available by the authors without undue reservation.
