# Supplementary material for: Potential inappropriate medications and drug–drug interactions in adverse drug reactions in the elderly: a retrospective study in a pharmacovigilance database
Source: Front Pharmacol. 2025 Apr 8;16:1546012. doi: 10.3389/fphar.2025.1546012 (PMC12011604; doi:10.3389/fphar.2025.1546012)
Supplement: Supplementary file 1 [file Table1.docx]

Supplementary Material

Table 1 Prescriptions revealed by the Beers criteria 2023

| Criteria | Organ system, therapeutic category, main active ingredient(s) (**Frequency**) | **Number of PIMs** (Total=692) |
| --- | --- | --- |
| **Potentially inappropriate medications use in Older Adults (n=311)** | | |
| **Antihistamines** | First-generation antihistamines: Chlorphenamine (58), [Promethazine](https://db.yaozh.com/atc?atc_num=R06AD52) (9), [Dimenhydrinate](https://db.yaozh.com/atc?atc_num=R06AA11) (1) | 68 |
| **Cardiovascular and antithrombotic** | Warfarin (25), Rivaroxaban (30), Dipyridamole (3), [Doxazosin](https://db.yaozh.com/atc?atc_num=C02CA04) (3), [Terazosin](https://db.yaozh.com/atc?atc_num=G04CA03) (1), [Clonidine](https://db.yaozh.com/atc?atc_num=C02AC01) (6), [Nifedipine](https://db.yaozh.com/atc?atc_num=C08CA05) (7), [Amiodarone](https://db.yaozh.com/atc?atc_num=C01BD01) (31), [Digoxin](https://db.yaozh.com/atc?atc_num=C01AA05) (7) | 113 |
| **Central nervous system** | Antidepressants with strong anticholinergic activity: [Doxepin](https://db.yaozh.com/atc?atc_num=D04AX01) (1) | 1 |
|  | Antiparkinsonian with strong anticholinergic activity: [Trihexyphenidyl](https://db.yaozh.com/atc?atc_num=N04AA01) (1) | 1 |
|  | First- (typical) generation antipsychotics: [Haloperidol](https://db.yaozh.com/atc?atc_num=N05AD01) (1), [Perphenazine](https://db.yaozh.com/atc?atc_num=N05AB03) (1)  Second-(atypical) generation antipsychotics: [Quetiapine](https://db.yaozh.com/atc?atc_num=N05AH04) (1) | 3 |
|  | Barbiturates: [Phenobarbital](https://db.yaozh.com/atc?atc_num=N03AA02) (1) | 1 |
|  | Benzodiazepines: [Alprazolam](https://db.yaozh.com/atc?atc_num=N05BA12) (4), [Midazolam](https://db.yaozh.com/atc?atc_num=N05CD08) (1), [Estazolam](https://db.yaozh.com/atc?atc_num=N05CD04) (2), [Clonazepam](https://db.yaozh.com/atc?atc_num=N03AE01) (1) | 8 |
|  | Non-benzodiazepines: [Eszopiclone](https://db.yaozh.com/atc?atc_num=N05CF04) (1) | 1 |
| **Endocrine system** | Insulin (13) | 13 |
|  | Sulfonylureas: Sulfonylureas (3), Glimepiride (5) | 8 |
|  | [Levothyroxine](https://db.yaozh.com/atc?atc_num=H03AA01) (2) | 2 |
|  | [Megestrol](https://db.yaozh.com/atc?atc_num=G03AC05) (3) | 3 |
| **Gastrointestinal system** | Proton pump inhibitors were used for more than 8 weeks without special conditions: [Omeprazole](https://db.yaozh.com/atc?atc_num=A02BC01) (5), Llaprazole (1), [Lansoprazole](https://db.yaozh.com/atc?atc_num=A02BC03) (3), [Rabeprazole](https://db.yaozh.com/atc?atc_num=A02BC04) (3), [Pantoprazole](https://db.yaozh.com/atc?atc_num=A02BC02) (1) | 13 |
|  | GI antispasmodics with strong anticholinergic activity: Atropine (1) | 1 |
|  | Desmopressin (1) | 1 |
|  | Non-COX-2-selective NSAIDs: Indomethacin (1), [Diclofenac](https://db.yaozh.com/atc?atc_num=D11AX18) (45), Meloxicam (3), Flurbiprofen (3), Ibuprofen (19), Piroxicam (2) | 73 |
|  | Skeletal muscle relaxants: Chlorzoxazone (1) | 1 |
| **Potentially inappropriate medication use in older adults due to drug–disease or drug–syndrome interactions that may exacerbate the disease or syndrome** **(n=194)** | | |
| **Heart failure** | Dextromethorphan (3), [Cilostazol](https://db.yaozh.com/atc?atc_num=B01AC23) (3), [Diltiazem](https://db.yaozh.com/atc?atc_num=C05AE03) (6) | 12 |
| **Syncope** | [Doxazosin](https://db.yaozh.com/atc?atc_num=C02CA04) (1) | 1 |
| **History of gastric or duodenal ulcer using a non-COX-2 selective NSAIDs** | Aspirin (97), [Piroxicam](https://db.yaozh.com/atc?atc_num=M01AC01) (1), Ibuprofen (13), [Paracetamol](https://db.yaozh.com/atc?atc_num=N02AJ06) (19), [Diclofenac](https://db.yaozh.com/atc?atc_num=D11AX18) (24) | 154 |
| **History of falls or fractures** | [Alprazolam](https://db.yaozh.com/atc?atc_num=N05BA12) (4), [Trihexyphenidyl](https://db.yaozh.com/atc?atc_num=N04AA01) (1), [Valproic Acid](https://db.yaozh.com/atc?atc_num=N03AG01) (8), [Perphenazine](https://db.yaozh.com/atc?atc_num=N05AB03) (1), [Caffeine](https://db.yaozh.com/atc?atc_num=D11AX26) (1), [Dihydrocodeine](https://db.yaozh.com/atc?atc_num=N02AA08) (4), [Codeine](https://db.yaozh.com/atc?atc_num=N02AJ06) (2), [Citalopram](https://db.yaozh.com/atc?atc_num=N06AB04) (4), [Eszopiclone](https://db.yaozh.com/atc?atc_num=N05CF04) (1) | 26 |
| **Dementia or cognitive impairment** | [Clonazepam](https://db.yaozh.com/atc?atc_num=N03AE01) (1) | 1 |
| **Drugs to be used with caution in older adults (n=146)** | | |
|  | [Ticagrelor](https://db.yaozh.com/atc?atc_num=B01AC24) (4) | 4 |
| **Antidepressants used with caution in the elderly** | SNRIs: [Venlafaxine](https://db.yaozh.com/atc?atc_num=N06AX16) (3)  SSRIS: [Fluoxetine](https://db.yaozh.com/atc?atc_num=N06AB03) (1), [Citalopram](https://db.yaozh.com/atc?atc_num=N06AB04) (6)  TCGS: [Duloxetine](https://db.yaozh.com/atc?atc_num=N06AX21) (1), [Doxepin](https://db.yaozh.com/atc?atc_num=D04AX01) (1), [Trazodone](https://db.yaozh.com/atc?atc_num=N06AX05) (2) | 14 |
|  | [Sulfamethoxazole and Trimethoprim](https://db.yaozh.com/atc?atc_num=J01EE01) (2) | 2 |
| **Antiepileptics** | [Oxcarbazepine](https://db.yaozh.com/atc?atc_num=N03AF02) (1) | 1 |
| **Diuretics** | [Furosemide](https://db.yaozh.com/atc?atc_num=C03CA01) (9), [Torasemide](https://db.yaozh.com/atc?atc_num=C03CA04) (16), [Hydrochlorothiazide](https://db.yaozh.com/atc?atc_num=C03AA03) (38), [Spironolactone](https://db.yaozh.com/atc?atc_num=C03DA01) (12), [Triamterene](https://db.yaozh.com/atc?atc_num=C03DB02) (1) | 76 |
|  | [Tramadol](https://db.yaozh.com/atc?atc_num=N02AX02) (1) | 1 |
|  | [Dextromethorphan](https://db.yaozh.com/atc?atc_num=R05DA09) (35) | 35 |
| **Sodium-glucose cotransporter-2 (SGLT2) inhibitors** | [Dapagliflozin](https://db.yaozh.com/atc?atc_num=A10BK01) (13) | 13 |
| **Drug-drug interactions that should be avoided in older adults (n=3)** | | |
| **≥3 CNS active drug combinations** | [Eszopiclone](https://db.yaozh.com/atc?atc_num=N05CF04) (1), [Trazodone](https://db.yaozh.com/atc?atc_num=N06AX05) (1), [Duloxetine](https://db.yaozh.com/atc?atc_num=N06AX21) (1) | 3 |
| **Medications that should be avoided or have their dosage reduced with varying levels of kidney function in older adults (n=38)** | | |
|  | CrCl < 30mL/min, Enoxaparin did not reduce dose (2) | 2 |
|  | CrCl < 30mL/min, NSAIDs (non-selective, COX-2 selective and non-acetylated salicylate, oral and parenteral) should be avoided but was used：Aspirin (9), [Celecoxib](https://db.yaozh.com/atc?atc_num=L01XX33) (2), [Diclofenac](https://db.yaozh.com/atc?atc_num=D11AX18) (2), [Paracetamol](https://db.yaozh.com/atc?atc_num=N02BE01) (2) | 15 |
|  | 15＜CrCl＜50mL/min, [Edoxaban](https://db.yaozh.com/atc?atc_num=B01AF03) did not reduce dose (1) | 1 |
|  | CrCl＜30mL/min, [Duloxetine](https://db.yaozh.com/atc?atc_num=N06AX21) should be avoided but was used (1) | 1 |
|  | CrCl＜30mL/min, [Sulfamethoxazole and Trimethoprim](https://db.yaozh.com/atc?atc_num=J01EE01) did not reduce dose (1) | 1 |
|  | CrCl＜15mL/min, Rivaroxaban should be avoided but was used (3) | 3 |
|  | CrCl＜30mL/min, Spironolactone should be avoided but was used (4) | 4 |
|  | CrCl＜30mL/min, Colchicine did not reduce dose (10) | 10 |
|  | CrCl＜30mL/min, Tramadol (Immediate release) did not reduce dose (1) | 1 |

Table 2 Serious ADRs caused by PIMs identified in ADRs reports

| **Main active ingredient(s) (**Frequency**)** | **Risk factors for PIMs（Total PIMs = 149）** | **Reported serious ADRs (**Frequency**)** |
| --- | --- | --- |
| Aspirin (57) | History of gastric and duodenal ulcers with aspirin or non-COX-2-selective NSAID (56);  CrCl < 30mL/min increased acute kidney injury risk (1) | Gastrointestinal bleeding(49), melena (14), hematemesis (3), nonspecific hemorrhage, peptic ulcer bleeding, abnormal renal function |
| [Diclofenac](https://db.yaozh.com/atc?atc_num=D11AX18) (23) | Potentially inappropriate medications use in older adults (23);  History of gastric and duodenal ulcers with aspirin or non-COX-2-selective NSAID (14) | Gastrointestinal bleeding (15), gastric ulcer bleeding, melena (10), nonspecific hemorrhage, peptic ulcer, peptic ulcer bleeding |
| Ibuprofen (9) | Potentially inappropriate medications use in older adults (9);  History of gastric and duodenal ulcers with aspirin or non-COX-2-selective NSAID (7) | Gastrointestinal bleeding (8), hematemesis, melena |
| [Paracetamol](https://db.yaozh.com/atc?atc_num=N02AJ06) (8) | History of gastric and duodenal ulcers with aspirin or non-COX-2-selective NSAID (8) | Gastrointestinal bleeding (7), melena (4), hematemesis |
| Warfarin (6) | Potentially inappropriate medications use in older adults (6) | Gastrointestinal bleeding (3), coagulation disorder, nonspecific hemorrhage (2) |
| Rivaroxaban (6) | Potentially inappropriate medications use in older adults (6);  CrCl＜15mL/min, rivaroxaban should be avoided but was used (2) | Gastrointestinal bleeding (3), melena (3), nonspecific hemorrhage, hematuria |
| [Ticagrelor](https://db.yaozh.com/atc?atc_num=B01AC24) (3) | Caution for the elderly (3) | Gastrointestinal bleeding (2), coagulation disorder |
| [Piroxicam](https://db.yaozh.com/atc?atc_num=M01AC01) (2) | Potentially inappropriate medications use in older adults (2);  History of gastric and duodenal ulcers with aspirin or non-COX-2-selective NSAID (1) | Gastrointestinal bleeding, peptic ulcer, peptic ulcer bleeding |
| Meloxicam (2) | Potentially inappropriate medications use in older adults (2) | Gastrointestinal bleeding, acute renal failure |
| [Estazolam](https://db.yaozh.com/atc?atc_num=N05CD04) (1) | Potentially inappropriate medications use in older adults (1) | Delirium, lethargy, dyspnea |
| [Dextromethorphan](https://db.yaozh.com/atc?atc_num=R05DA09) (1) | Caution for the elderly (1) | Giddy |
| Desmopressin (1) | Potentially inappropriate medications use in older adults (1) | Edema, electrolyte anomaly |
| [Digoxin](https://db.yaozh.com/atc?atc_num=C01AA05) (1) | Avoid inappropriate medication for the elderly in patients with heart failure (1) | Disgusting, omiting, bradycardia |
| [Haloperidol](https://db.yaozh.com/atc?atc_num=N05AD01) (1) | Potentially inappropriate medications use in older adults (1) | Tardive dyskinesia |
| [Hydrochlorothiazide](https://db.yaozh.com/atc?atc_num=C03AA03)(1) | Caution using diuretics for elderly (1) | Hyponatremia |
| [Celecoxib](https://db.yaozh.com/atc?atc_num=L01XX33) (1) | CrCl < 30mL/min increased acute kidney injury risk (1) | Abnormal renal function |
| Enoxaparin (1) | CrCl < 30mL/min increases bleeding risk (1) | Gastrointestinal bleeding |
| Human insulin (1) | Potentially inappropriate medications use in older adults (1) | Hypoglycemic coma |

Table 3 Serious ADRs caused by pDDIs identified in ADRs reports

| **Drug pairs** | **n** | **Risk rating** | **Reliability of pDDIs** | **Potential clinical consequences** | **Reported ADRs** | **Mechanism of interaction** |
| --- | --- | --- | --- | --- | --- | --- |
| Aspirin-clopidogrel | 17 | C | Fair | Enhanced antiplatelet drugs adverse/toxic effects and increased bleeding risk | Gastrointestinal bleeding(15), hematuria(2), melena(4), gingival bleeding, epistaxis | PD |
| Diclofenac-ginseng | 6 | C | Fair | Increased bleeding risk | Melena(5), nonspecific hemorrhage, gastrointestinal bleeding(2) | PD |
| Aspirin-ginseng | 4 | C | Fair | Increased bleeding risk | Gastrointestinal bleeding(4) | PD |
| Clopidogrel-diclofenac | 3 | C | Fair | Enhanced antiplatelet drugs adverse/toxic effects and increased bleeding risk | Melena(3) | PD |
| Bisoprolol-diltiazem | 2 | C | Good | Enhanced the bradycardic effect | Bradycardia(2) | PD |
| Heparin-ginkgo biloba | 2 | C | Fair | Enhanced anticoagulant effect and increased bleeding risk | Gastrointestinal bleeding, hematuria, gingival bleeding, epistaxis, melena | PD |
| Clopidogrel-ginkgo biloba | 2 | C | Fair | Enhanced antiplatelet drugs adverse/toxic effects and increased bleeding risk | Gastrointestinal bleeding, gingival bleeding, epistaxis, hematuria, melena | PD |
| Diclofenac-losartan potassium and hydrochlorothiazide | 2 | C | Good | Increased nephrotoxicity | Melena(2) | PD |
| Diclofenac-ginkgo biloba | 2 | C | Fair | Enhanced antiplatelet drugs adverse/toxic effects and increased bleeding risk | Gastrointestinal bleeding(2) | PD |
| Promethazine-reserpine | 2 | C | Fair | Enhanced CNS adverse/toxic effect | Celialgia, anorexia, gastrointestinal bleeding | PK/PD |
| Aacarbose-repaglinide | 1 | C | Fair | Enhanced hypoglycemic effect | Hypoglycemic coma | PD |
| Aspirin-furosemide | 1 | C | Fair | Increase salicylates serum concentration and bleeding risk | Gastrointestinal bleeding | PK/PD |
| Aspirin-prednisolone | 1 | C | Good | Enhanced corticosteroids adverse/toxic effect and increased bleeding risk | Gastrointestinal bleeding | PK/PD |
| Aspirin-tirofiban | 1 | C | Good | Enhanced antiplatelet drugs adverse/toxic effects and increased bleeding risk | Gastrointestinal bleeding | PK/PD |
| Aspirin-ethanol | 1 | C | Fair | Increased bleeding risk | Gastrointestinal bleeding | PD |
| Edoxaban-ginseng | 1 | C | Fair | Enhanced anticoagulants effects and increased bleeding risk | Cerebral hemorrhage | PD |
| Benazepril-torasemide | 1 | C | Good | Enhanced antihypertensive effect and increased nephrotoxicity | Abnormal renal function | PD |
| Ibuprofen-olmesartan | 1 | C | Good | Enhanced nonsteroidal anti-Inflammatory agents adverse/toxic effect and increased nephrotoxicity | Gastrointestinal bleeding | PK/PD |
| Methylprednisolone-aspirin | 1 | C | Fair | Enhance nonsteroidal anti-inflammatory agents adverse/toxic effect and increased bleeding risk | Hematemesis, gastrointestinal bleeding | PK/PD |
| Ritonavir-betamethasone | 1 | C | Fair | Increased betamethasone serum concentration | Abnormal liver function | PK |
| Ritonavir-methylprednisolone | 1 | C | Excellent | Increased methylprednisolone serum concentration | Abnormal liver function | PK |
| Loxoprofen-methylprednisolone | 1 | C | Good | Enhanced corticosteroids adverse/toxic effect and increased bleeding risk | Hematemesis, gastrointestinal bleeding | PK/PD |
| Ginseng-clopidogrel | 1 | C | Fair | Enhanced antiplatelet drugs adverse/toxic effects and increased bleeding risk | Melena | PD |
| Rosuvastatin-clopidogrel | 1 | C | Good | Increased rosuvastatin serum concentration | Gastrointestinal bleeding, melena | PK/PD |
| Celecoxib-ginseng | 1 | C | Fair | Increased bleeding risk | Gastrointestinal bleeding | PD |
| Diclofenac-candesartan | 1 | C | Good | Enhanced nonsteroidal anti-inflammatory agents adverse/toxic effect and increased nephrotoxicity | Gastrointestinal bleeding | PD |
| Diclofenac-valsartan | 1 | C | Good | Increased nephrotoxic effect | Liver cirrhosis | PD |
| Diclofenac-ethanol | 1 | C | Fair | Increased bleeding risk | Gastrointestinal bleeding, melena | PD |
| Tirofiban-ticagrelor | 1 | C | Fair | Enhanced antiplatelet effect and increased bleeding risk | Gastrointestinal bleeding | PK/PD |
| Vancomycin-torasemide | 1 | C | Good | Increased nephrotoxic effect | Creatinine clearance decreased | PD |
| Vinorelbine-cisplatin | 1 | C | Fair | Enhanced granulocytopenia risk | Myelosuppression, convulsion | PD |
| Heparin-aspirin | 4 | D | Good | Enhanced anticoagulant effect | Gastrointestinal bleeding(3), gingival bleeding, epistaxis, hematuria, melena | PK/PD |
| Aspirin-diclofenac | 4 | D | Good | Increased bleeding risk | Gastrointestinal bleeding(2), giddy(2), fatigue(2), melena(2) | PK/PD |
| Aspirin-ibuprofen | 3 | D | Good | Increased bleeding risk | Gastrointestinal bleeding(3) | PK/PD |
| Aspirin-ginkgo biloba | 3 | D | Fair | Enhanced anticoagulant effect | Gastrointestinal bleeding(2), gingival bleeding, epistaxis, hematuria melena | PD |
| Heparin-clopidogrel | 3 | D | Good | Enhanced anticoagulant effect | Gastrointestinal bleeding(2), gingival bleeding, epistaxis, hematuria, melena | PD |
| Rivaroxaban-aspirin | 3 | D | Fair | Increased bleeding risk | Melena(2), hematuria, gastrointestinal bleeding | PD |
| Rivaroxaban-clopidogrel | 3 | D | Fair | Increased bleeding risk | Gastrointestinal system damage, hematuria, gastrointestinal bleeding, melena | PD |
| Aspirin-warfarin | 2 | D | Excellent | Enhanced anticoagulant effect | Gastrointestinal bleeding(2) | PD |
| Aspirin-loxoprofen | 2 | D | Good | Increased bleeding risk | Gastrointestinal bleeding(2), hematemesis(2), melena | PK/PD |
| Aspirin-ticagrelor | 3 | D | Fair | Enhanced antiplatelet effect and increased bleeding risk | Coagulation disorder, gastrointestinal bleeding (2), hematemesis | PD |
| Heparin-urokinase | 2 | D | Fair | Increased bleeding risk | Coagulation disorder, gastrointestinal bleeding | PD |
| Heparin-dipyridamole | 2 | D | Good | Enhanced anticoagulant effect | Gastrointestinal bleeding, gingival bleeding；epistaxis, hematuria, melena； | PD |
| Heparin-tirofiban | 2 | D | Good | Enhanced anticoagulant effect | Gastrointestinal bleeding, thrombopenia | PD |
| Rivaroxaban-diclofenac | 2 | D | Fair | Increased bleeding risk | Gastrointestinal bleeding, melena(2) | PD |
| Aspirin-celecoxib | 1 | D | Good | Enhanced nonsteroidal anti-inflammatory agents adverse/toxic effect | Gastrointestinal bleeding | PD |
| Estazolam-dexmedetomidine | 1 | D | Fair | Enhanced CNS depressant effect | Drowsiness | PD |
| Digoxin-amiodarone | 1 | D | Excellent | Increased digoxin serum concentration | Atrioventricular block, bradycardia, increased blood concentration | PK |
| Febuxostat-rosuvastatin | 1 | D | Good | Increased rosuvastatin serum concentration | Gastrointestinal bleeding, elevated liver enzymes | PK |
| Heparin-ticagrelor | 1 | D | Good | Increased bleeding risk | Gastrointestinal bleeding | PK/PD |
| Enoxaparin-aspirin | 1 | D | Fair | Increased bleeding risk | Gastrointestinal bleeding | PK/PD |
| Enoxaparin-urokinase | 1 | D | Fair | Increased bleeding risk | Gastrointestinal bleeding | PK/PD |
| Enoxaparin-ticagrelor | 1 | D | Fair | Increased bleeding risk | Gastrointestinal bleeding | PK/PD |
| Enoxaparin-tirofiban | 1 | D | Fair | Increased bleeding risk | Gastrointestinal bleeding | PK/PD |
| Paclitaxel-cisplatin | 1 | D | Fair | Enhanced myelosuppressive effect | Myelosuppression | PD |
| Potassium chloride-promethazine | 2 | X | Fair | Increased ulcerogenic and bleeding risk | Gastrointestinal bleeding, celialgia | PK/PD |
| Urokinase-tirofiban | 1 | X | Fair | Enhanced anticoagulant effect | Gastrointestinal bleeding | PK/PD |
| Urokinase-ticagrelor | 1 | X | Fair | Enhanced anticoagulant effect | Gastrointestinal bleeding | PK/PD |
| Urokinase-aspirin | 1 | X | Fair | Enhanced anticoagulant effect | Gastrointestinal bleeding | PK/PD |
| Potassium chloride-chlorphenamine | 1 | X | Fair | Increased ulcerogenic and bleeding risk | Gastrointestinal bleeding | PK/PD |
| Dexamethasone-desmopressin | 1 | X | Fair | Increased hyponatremic risk | Electrolyte anomaly | PD |

**
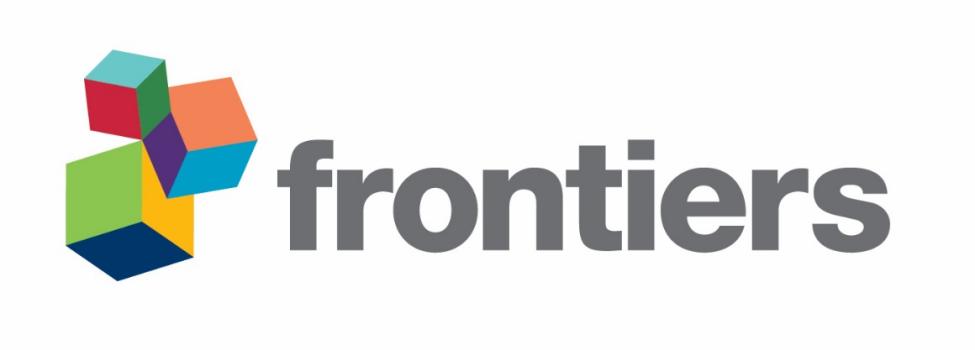
**

**Supplementary Figure 1.** The figure legends are required to have the same font as the main text, 12 point normal Times New Roman, single spaced. Please use a single paragraph for each legend and prepare the figures keeping in mind the PDF layout.
